# Environmental Surveillance of Genogroup I and II Noroviruses in Shandong Province, China in 2013

**DOI:** 10.1038/srep17444

**Published:** 2015-11-30

**Authors:** Zexin Tao, Minglei Xu, Xiaojuan Lin, Haiyan Wang, Lizhi Song, Suting Wang, Nan Zhou, Dongfeng Zhang, Aiqiang Xu

**Affiliations:** 1 Academy of Preventive Medicine, Shandong University, Jinan, People’s Republic of China; 2 Shandong Provincial Key Laboratory of Infectious Disease Control and Prevention, Shandong Center for Disease Control and Prevention, Jinan, People’s Republic of China; 3 Qingdao Medical College, Qingdao University, No. 38 Dengzhou Road Qingdao, 266021, china; 4 School of Public Health, Shandong University, Jinan, People’s Republic of China

## Abstract

Noroviruses are the most common cause of epidemic gastroenteritis. However, the case-based surveillance is limited in China. In this study, we analyzed the results of environmental surveillance conducted in two cities of Shandong Province, China from January to December in 2013. Twenty-four sewage samples were collected and concentrated via membrane absorption/elution method. After reverse transcription-PCR, cloning and sequencing on ORF2 region, norovirus nucleic acid was detected in all 24 sewage samples. A total of 403 norovirus sequences of 16 genotypes were detected, among which GII.3 (22.6%), GI.2 (17.1%), GI.5 (13.4%), GI.3 (11.9%), GII.4 (7.7%), and GII.6 (6.7%) were the 6 most common genotypes. Phylogenetic analysis revealed multiple lineages within most common genotypes, especially in GI.3, whereas all GII.4 sequences belonged to Sydney 2012 strain. Recombination events were observed in 5 GI and 4 GII sequences within or near the ORF1/ORF2 overlap. This is the first report on systematic environmental surveillance on norovirus in China. The data presented here reveal co-circulation and high genetic diversity of multiple norovirus genotypes in the two cities, and suggest continued environmental surveillance can provide valuable information on norovirus circulation in the population.

Noroviruses form a genus in the family *Caliciviridae*. They are the most common cause of acute gastroenteritis in people of all ages. Its genome is a single positive-strand RNA approximately 7.7 kb in size containing three open reading frames (ORFs) that encode nonstructural (ORF1) and structural proteins (ORF2, coding for VP1, and ORF3, coding for VP2)^1^.

The diversity among noroviruses is great, and they were classified on the basis of their sequences into 6 genogroups^1^. Noroviruses of genogroup I (GI), GII, and GIV are responsible for disease in humans and they contain at least 32 genotypes^1^,^2^. Outbreaks caused by GI and GII viruses were frequently reported throughout the world, whereas GIV viruses were detected as the cause of only a handful of outbreaks in humans. A single genotype of GII.4 has been the predominant cause of major acute gastroenteritis outbreaks and epidemics in many countries (including China) since the mid-1990s^3^. It is estimated to be responsible for 60 to 80 percent of all norovirus-associated outbreaks worldwide^4^,^5^.

Fecal–oral spread is the primary transmission mode of noroviruses. They are shed in the feces of infected individual, and can be detected in sewage samples. Hence, detecting noroviruses in the sewage could reflect the actual status of viral circulation in the area. Previous reports have shown that norovirus sequences detected in sewage were closely related to those from gastroenteritis cases in an outbreak^6^, and revealed the existence of continuous circulation of some genotypes in sewage that seldom detected from gastroenteritis patients^7^.

In China, noroviruses were frequently reported to be associated with gastroenteritis outbreaks, especially in autumn/winter seasons. Scattered reports had described the molecular epidemiology of noroviruses from clinical gastroenteritis patients in specific areas of China. According to these studies, genotypes of GII was frequently detected, but GI was seldom seen^8^,^9^,^10^. Regarding the norovirus presence in sewage in China, no information was available except for a one year survey conducted monthly in three sewage treatment plants in Beijing^11^, while the results showed that norovirus was detected with a extremely low frequency (3.1%), which is quite different from similar studies in other countries^7^,^12^,^13^.

Enterovirus environmental surveillance has been conducted in our laboratory since 2008. Previous reports have revealed the correlation between environmental enterovirus strains and those from clinical patients, and demonstrated the high sensitivity of the surveillance in tracing intercity spread of a certain lineage^14^,^15^,^16^. To better understand the molecular epidemiology of norovirus in China, we describe and analyze circulation and genotypes of GI and GII noroviruses from sewage in Shandong Province in 2013.

## Results

### Genotype profiles

A total of 24 sewage samples were collected in the cities of Jinan and Linyi from January to December in 2013. RT-PCR was performed on concentrated sewage solution and norovirus GI and GII sequences were detected in all 24 samples. After TA cloning of PCR products and sequencing, altogether 480 insert sequences were obtained. Among these, 77 sequences were identified to be bacterial sequences from nonspecific amplification, and the rest 403 were norovirus sequences (220 GI and 183 GII). They belonged to 16 genotypes including GI.1, GI.2, GI.3, GI.4, GI.5, GI.6, GI.8, GI.9, GII.2, GII.3, GII.4, GII.6, GII.8, GII.13, GII.17, and GII.21 (Table 1). Among GI genotypes, GI.2, GI.5, GI.3 were the 3 most common genotypes accounting for 31.4% (69/220), 24.5% (54/220), and 21.8% (48/220) respectively of total GI detection. GII.3 (49.7%, 91/183), GII.4 (16.9%, 31/183), and GII.6 (14.8%, 27/183) were the 3 most common GII genotypes. The two most common genotypes, GI.2 and GII.3, were observed in almost every month during the study period, suggesting the continuous circulation in the population. GII.4, the most prevalent cause of acute gastroenteritis epidemic throughout the world, only accounted for 16.9% of total GII detection, and was detected in 70.8% (17/24) of sewage samples. GI.6, GI.8, GI.9, GII.2, GII.8, GII.13, and GII.21 were detected with a low frequency (altogether 4.96% of total norovirus detection), suggesting they were minor circulating viruses. No dramatic difference on genotypes composition was observed between the two cities.

### Sequence analysis of GI noroviruses

**Figure 1:**
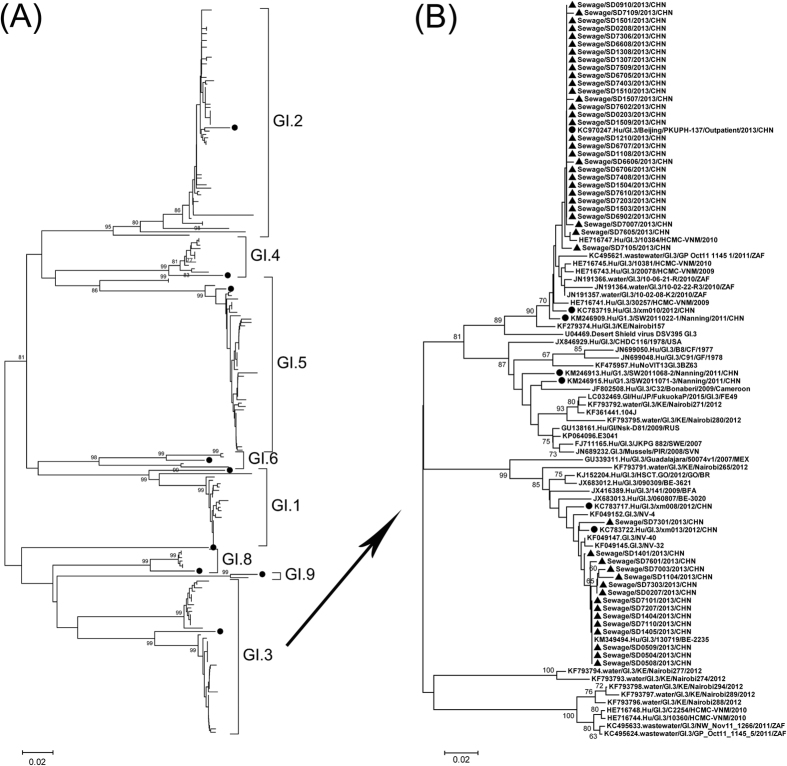
Phylogenetic relationships of GI (A) and GI.3 (B) noroviruses detected in sewage in Shandong, 2013. The phylogenetic trees were constructed via Mega 4.0, using the NJ method based on 314-nt GI (positions 5358 to 5671 on strain Norwalk/68/US) partial VP1 sequences. ● reference strains of each genotypes. Other branches in A and ▲ in B indicate Shandong sequences from sewage. GI.3 branches in A are enlarged to B with addition of GI.3 sequences from other countries available in GenBank.

Homologous comparison and phylogenetic analysis of GI noroviruses among Shandong and reference strains were conducted based on 314-nt GI (positions 5358 to 5671 on strain Norwalk/68/US) partial VP1 sequences. Shandong sequences within most genotypes had high similarity with each other (Table 2), whereas high range of genetic diversity was observed in the genotypes of GI.3, GI.5, and GI.6. Phylogenetic relationship of Shandong strains with reference strains are illustrated in Fig. 1A. Multiple lineages were observed in most genotypes, suggesting local co-circulation of multiple transmission lineages.

GI.3 sequences from sewage in this study were phylogenetically compared with those from all over the world available in GenBank (Fig. 1B). Two distinct clusters of Shandong sequences were observed in the VP1 tree. In the major cluster, Shandong sequences had close relationship with those from Vietnam, South Africa, and other 3 Chinese cities of Beijing, Xiamen and Nanning from 2009 to 2013. In the minor cluster, Shandong sequences had close relationship with those from USA, Burkina Faso, Belgium, Thailand, and the city of Xiamen from 2011 to 2013.

### Sequence analysis of GII noroviruses

**Figure 2:**
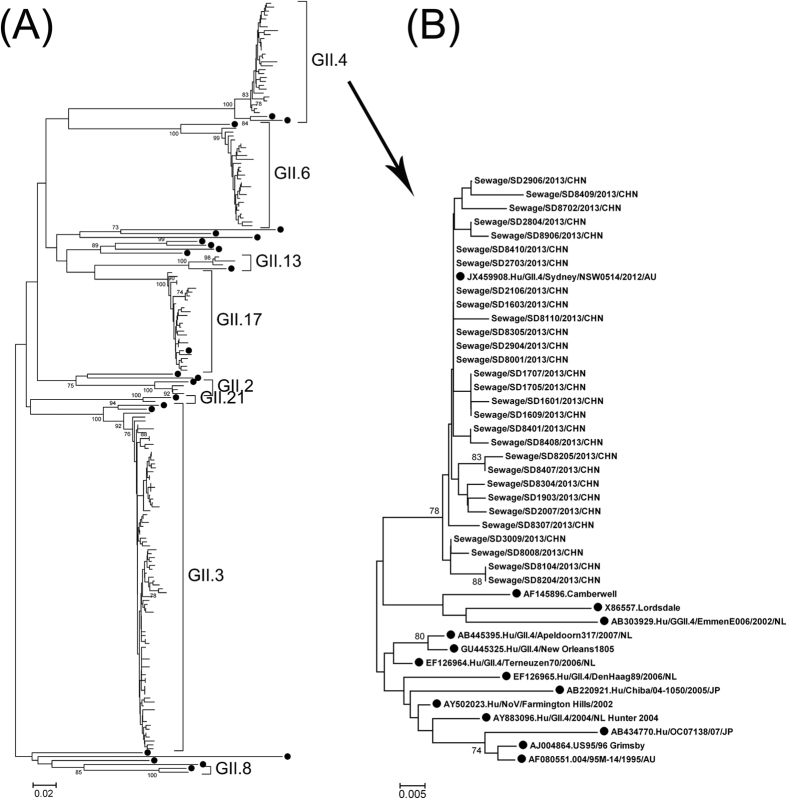
Phylogenetic relationships of GII (A) and GII.4 (B) noroviruses detected in sewage in Shandong, 2013. The phylogenetic trees were constructed via Mega 4.0, using the NJ method based on 305-nt GII (positions 5085 to 5389 on strain Lordsdale/93/UK) partial VP1 sequences. ● reference strains of each genotypes. Other branches indicate Shandong sequences from sewage. GII.4 branches in A are enlarged to B with addition of GII.4 reference sequences.

Shandong GII noroviruses from sewage were compared with reference strains based on 305-nt GII (positions 5085 to 5389 on strain Lordsdale/93/UK) partial VP1 sequences. Shandong sequences within most genotypes had high similarity with each other except for GII.3 (Table 2). Phylogenetic relationship of Shandong strains with reference strains are illustrated in Fig. 2A. Also, co-circulation of multiple transmission lineages was observed.

Shandong GII.4 sequences were phylogenetically compared with those variants from previous major epidemics from 1995 to 2012, including US95/96, Farmington Hills 2002, Hunter 2004, Yerseke 2006a and Den Haag 2006b, Apeldoorn 2008, New Orleans 2010, and Sydney 2012 (21). All Shandong sequences from sewage in 2013 were grouped into a cluster of Sydney 2012 (Fig. 2B). The mean *p*-distance between Shandong sequences and the Sydney 2012 strain was 0.0042.

### Recombination

**Figure 3:**
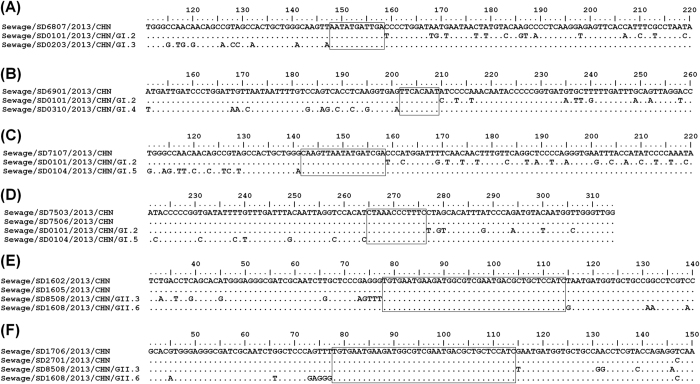
Recombination in 5 GI and 4 GII norovirus sequences from sewage. Evidences of recombination are observed in the sequences of SD6807 (**A,** GI.2/GI.3), SD6901 (**B,** GI.2/GI.4), SD7107 (**C,** GI.2/GI.5), SD7503 (**D,** GI.2/GI.5), SD7506 (**D,** GI.2/GI.5), SD1602 (**E,** GII.6/GII.3), SD1605 (**E,** GII.6/GII.3), SD1706 (**F,** GII.3/GII.6) and SD2701 (**F,** GII.3/GII.6). Sequences in the boxes indicate crossover sites. Each recombination sequences are aligned with two local parental sequences with no evidence of recombination in comparison with reference strains of each genotype. Nucleotide positions are indicated above the sequences. The 1^st^ position in (**A**) to (**D**) corresponds to genomic position 5342 of strain Norwalk/68/US (accession no., M87661), and the 1^st^ position in (**E,F**) corresponds to genomic position 5003 of strain Lordsdale/93/UK (accession no., X86557).

In alignment of the 314-nt GI capsid sequences, recombination events were observed in 5 sequences (2.3%, 5/220) from sewage, i.e. SD6807 (GI.2/GI.3), SD6901 (GI.2/GI.4), SD7107 (GI.2/GI.5), SD7503 (GI.2/GI.5) and SD7506 (GI.2/GI.5) (Fig. 3A–D). Interestingly, GI.2 sequences participated in all recombination events. The crossover sites ranged from position 5483 to 5617 according to the genomic sequence of strain Norwalk/68/US (M87661), located downstream nearby ORF1/ORF2 junction.

In alignment of the 387-nt GII sequences, recombination events were observed in 4 sequences (2.2%, 4/183) including SD1602 (GII.6/GII.3), SD1605 (GII.6/GII.3), SD1706 (GII.3/GII.6), and SD2701 (GII.3/GII.6) (Fig. 3E,F). The crossover sites ranged from position 5080 to 5116 according to strain Lordsdale/93/UK, comprising the whole ORF1/ORF2 junction region.

## Discussion

This study presents an overview of norovirus genotypes and phylogeny in sewage in China. Currently, the case-based surveillance on norovirus in China is limited. Except for scattered investigation on related gastroenteritis outbreaks, no detailed information is available on the genotype spectrum and continuous transmission in human populations. In the Global Polio Eradication Initiative (GPEI), environmental surveillance is regarded as an effective approach for monitoring polioviruses, especially in regions with poor case-based surveillance^17^,^18^. Similarly, surveillance on domestic sewage will also be of great importance in understanding the local norovirus circulation.

Since norovirus copies in sewage are not as high as those in specimens from infected individuals, a concentration step has to be included in the sewage sample processing. Multiple methods are currently used in concentrating enteric viruses from sewage, and besides the membrane absorption/elution method used in this study, polyethylene glycol (PEG) precipitation, two-phase separation, ultracentrifugation, and various other methods are also widely used in global laboratories. These methods might differ in sensitivity, but no systematic comparative studies have been reported^18^. Iwai *et al.* used two methods in parallel to detect norovirus in sewage in Japan from 2006 to 2008, and found that membrane absorption/elution method was more sensitive^7^. In this study, although we did not compare the sensitivity with other methods, norovirus was detected in all sewage samples in the two cities throughout 2013, further demonstrating that membrane absorption/elution method is very efficient in concentrating enteric viruses. Furthermore, the 100% positive results suggest that the 3.1% positive rate in a previous study conducted in Beijing^11^ can not reflect the actual status of norovirus circulation, and the silica method used in that study may not be appropriate for concentrating noroviruses.

The data presented in this study reveal co-circulation and high genetic diversity of multiple norovirus genotypes in the two cities. A total of 16 genotypes were identified during the study period. GII.3, GI.2, GI.5, GI.3 were the 4 most common genotypes accounting for 65.0% (262/403) of total detection, suggesting these are predominantly circulating genotypes in the two cities in 2013. Generally, GI noroviruses are regarded as a relatively uncommon group, and systematic descriptions of GI outbreak epidemiology and characteristics are scarce^19^. But according to the norovirus surveillance system in the United States, an increase in the proportion of genotype GI.6 norovirus outbreaks in 2012 was observed^20^. So, the detection of various GI viruses in this study suggests GI gastroenteritis is an emerging concern in current China, although they may have slightly lower hospitalization rate and less severe outcomes compared to GII.4^20^.

As a predominant cause of global epidemics, GII.4 only accounted for 16.9% (31/183) of GII detection. This differs dramatically from the results of other similar studies conducted in Japan, Italy, Luxembourg and the Netherlands in which GII.4 was the most frequently detected genotype in sewage^7^,^21^,^22^,^23^. The relative lower constituent ratio of GII.4 and predominance of GII.3 observed in this study suggests a different GII genotype spectrum in local populations in 2013. It has been observed that new GII.4 strains have emerged every 2–3 years and replaced previously strains during the past decade^3^,^24^. All GII.4 sequences in this study belonged to Sydney 2012, revealing that it is a predominant GII.4 strain in Shandong in 2013. Since this is just a one-year study, continuous surveillance in the future will provide valuable information on the temporal fluctuation of various genotypes.

Noroviruses are a diverse group of pathogens and more and more new genotypes have been identified. In this study, some rare genotypes, such as GI.9 and GII.21, were also detected. The first record of observation of GI.9 in GenBank dates back to 2002 in Korea^25^. Subsequently, it is reported in many other countries such as Sweden^26^, Nigeria^27^, Kenya^28^, Thailand^29^, Singapore^30^, South Africa^31^, Jordan^32^, Egypt^33^, etc. Although these genotypes maintain rare in various surveillance reports, the possibility of their emergence as important causes of outbreaks cannot be excluded.

Recombination is a driving mechanism of norovirus evolution. Co-circulation of different norovirus genotypes leads to co-infection in humans, provides the possibility of recombination events, and gives rise to the circulation of new recombinant strains^34^. The highly conserved ORF1/ORF2 overlap is demonstrated to be a recombination hotspot^35^,^36^. In this study, 4 recombination sequences (2.2%) of GII viruses were observed in ORF1/ORF2 overlap. They were all GII.3 and GII.6 recombinants. For GI viruses, since amplified GI sequences in this study belonged to ORF 2 region, and no sequence of upstream viral RNA-dependent RNA polymerase (RdRp) region was obtained. So, the recombination events within the ORF1/ORF2 overlap in GI viruses cannot be identified. However, 5 recombination events (2.3%) were still observed in the downstream region nearby ORF1/ORF2 overlap. The >2% ratios in GI and GII norovirus in this study suggest high recombination activity, and further researches on circulating genotypes are essential to understand the origin and dynamics of the recombination events.

In conclusion, noroviruses are the leading cause of epidemic gastroenteritis. In the context of limited case-based surveillance, continued environmental surveillance will provide valuable information on viral circulation and enable further assessment of the early warning of emergence of relatively rare noroviruses.

## Methods

### Sampling

The permission for both sampling locations was issued by Shandong Provincial Environmental Protection Department. Sewage samples were collected monthly at the inlet collector canals of the wastewater treatment plants (WTPs) in the cities of Jinan and Linyi of Shandong Province from January to December in 2013. Both WTPs cover an area with about 800,000 inhabitants and the average inflows of raw sewage per day were about 6–10 × 10^4^ m^3^ derived from domestic sewage and industrial wastewater. Approximately 1.6 liters of sewage was collected by grab sampling method between 2:00 and 3:00 p.m. Cold temperature (approximately 4 °C) was maintained during sample transport to the laboratory, storage (<24 h), and processing.

### Concentration

Sewage samples were concentrated 80-fold via the membrane adsorption and elution method as described previously^14^,^15^,^16^. Briefly, 800 ml of the sewage was centrifuged at 3200 × *g* for 30 min at 4 °C. MgCl_2_ solution (2.5 M) was added to the supernatant to a final concentration of 0.05 M, and the pH was adjusted to 3.5 with hydrochloric acid (0.5 M). The solution was filtered through a 0.45 μm mixed cellulose ester membrane filter (ADVANTEC). Subsequently, the membrane was cut into small pieces and absorbents on the membrane were eluted with addition of 10 ml 3% beef extract solution followed by ultrasonication for 3 min. After centrifuge at 3 000 × *g* for 30 min, the supernatant was filtered through a 0.22 μm filter and was ready for RNA extraction.

### Reverse transcription-PCR

Total RNA was extracted from 1 milliliter of the concentrated sewage using High Pure Viral Nucleic Acid Large Volume Kit (Roche) according to the manufacturer’s procedure. Reverse transcription-PCR (RT-PCR) was performed using Access RT-PCR System (Promega, USA). Primer pair G1-SKF (CTG CCC GAA TTY GTA AAT GA) and G1-SKR (CCA ACC CAR CCA TTR TAC A) was used for amplification of a 330-nucleotide (nt) GI sequence corresponding to nt position 5342 to 5671 of strain Norwalk/68/US (accession no., M87661), and primer pair COG2F (CAR GAR BCN ATG TTY AGR TGG ATG AG) and G2-SKR (CCR CCN GCA TRH CCR TTR TAC AT) were used for amplification of a 387-nt GII sequence corresponding to nt position 5003 to 5389 of strain Lordsdale/93/UK (accession no., X86557). The amplified sequences encompass the 3′ end of ORF1 to the beginning of the capsid region^37^. In order to detect cross contamination, a RT-PCR reaction using the RNA extracted from 3% beef extract solution served as a blank control, and a negative control containing all the components of the reaction except for the template was also included.

### Cloning, sequencing and genotyping

RT-PCR products were analyzed by electrophoresis with 1.5% agarose gels. All positive products were gel-purified using a QIAquick Gel Extraction Kit (Qiagen) and were ligated into the pGEM®-T Easy vector (Promega) by TA cloning. The ligation products were transformed into competent *Escherichia coli* JM109 cells using the heat shock method. After blue and white screening, 10 positive recombinant clones were selected for each transformation, which meant theoretically 10 GI and 10 GII sequences could be obtained for each sewage sample. The plasmid was extracted and sequenced with a BigDye Terminator v3.1 Cycle Sequencing Kit (Applied Biosystems) and an ABI 3130 genetic analyzer (Applied Biosystems). Molecular typing based on partial capsid sequences was performed using online Norovirus Genotyping Tool version 1.0^38^.

### Homologous comparison and phylogenetic analysis

Nucleotide sequence alignments were conducted by BioEdit 7.0.5.3 software^39^. Phylogenetic analysis was performed by Mega 4.0^40^ using neighbor-joining method after estimation of genetic distance using the Kimura two-parameter method. A bootstrapping test was performed with 1,000 duplicates. The reference strains of GI and GII noroviruses used in this study are the following: GI.1 Norwalk/68/US M87661; GI.2 Southampton L07418; GI.3 DSV395 U04469; GI.4 Chiba407 AB022679; GI.5 Musgrove/89/UK AJ277614; GI.6 Kingston/ACT160D JQ388274; GI.7 Winchester/94 AJ277609; GI.8 Boxer/2001 AF538679; GI.9 R5/2010/Nigeria JN871684; GII.1 7EK/Hawaii/1971/USA JX289822; GII.2 Melksham X81879; GII.3 TV24 U02030; GII.3 HK46/1977/CHN KC597144; GII.4 Lordsdale X86557; GII.5 Hillingdon/90 AJ277607; GII.6 Seacroft/90/UK AJ277620; GII.7 Leeds/90 AJ277608; GII.8 Amsterdam/98-18 AF195848; GII.9 VA97207/1997 AY038599; GII.10 Erfurt/546/00/DE AF427118; GII.11 Sw918/1997/JP AB074893; GII.12 Wortley/90/UK AJ277618; GII.13 Fayetteville/1998/US AY113106; GII.14 M7/1999/US AY130761; GII.15 J23/1999/US AY130762; GII.16 Tiffin/1999/USA AY502010; GII.17 CS-E1/2002/USA AY502009; GII.18 swine/GII/OH-QW101/03/US AY823304; GII.19 swine/GII/OH-QW170/03/US AY823306; GII.21 Kawasaki/YO284 KJ196284. The accession numbers according to norovirus GII.4 capsid variants used in this study are the following: Camberwell 1994 AF145896; US95/96 AJ004864; AF080551; AB303929; Farmington Hills 2002 AY485642; Asia 2003 AB220921; Hunter 2004 AY883096; Terneuzen 2006a EF126964; Den Haag 2006b EF126965; AB434770; Apeldoorn AB445395; New Orleans 2010 GU445325; Sydney 2012 JX459908^24^.

## Additional Information

**Accession codes:** Nucleotide sequences determined in this study were deposited in the GenBank database under the accession numbers KR107558–KR107934.

**How to cite this article**: Tao, Z. *et al.* Environmental Surveillance of Genogroup I and II Noroviruses in Shandong Province, China in 2013. *Sci. Rep.* **5**, 17444; doi: 10.1038/srep17444 (2015).

## Figures and Tables

**Table 1:**
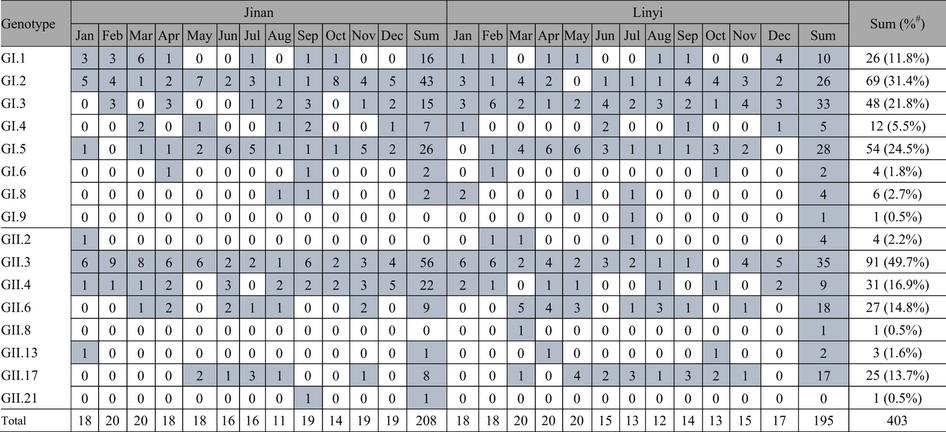
Numbers of Norovirus sequences detected in sewage in Shandong Province, 2013, by month and genotype*.

^*^Numbers other than zero are highlighted by grey shading. ^#^Constituent ratio within a genogroup.

**Table 2:** VP1 nucleotide identities among Shandong and reference sequences.

Genotypes	Nucleotide identity (%)	
	With reference strains*	Among Shandong sequences
GI.1	88.8–92.6	90.1–100.0
GI.2	93.6–98.0	94.9–100.0
GI.3	85.0–93.9	84.0–100.0
GI.4	94.2–95.5	97.1–100.0
GI.5	89.8–99.0	87.2–100.0
GI.6	86.9–94.2	85.0–99.6
GI.8	94.9–95.5	99.3–100.0
GI.9	97.1	NA#
GII.2	94.4–95.7	98.0–98.6
GII.3	88.8–94.4	89.8–100.0
GII.4	92.7–94.0	96.7–100.0
GII.6	91.4–92.7	96.7–100.0
GII.8	95.4	NA#
GII.13	93.1–94.7	97.7–98.3
GII.17	97.7–99.3	96.7–100.0
GII.21	96.7	NA#

^*^Reference strains refer to those listed in Materials and Methods section

^#^NA: Only one strain.
